# The Anti-Apoptotic and Cardioprotective Effects of Salvianolic Acid A on Rat Cardiomyocytes following Ischemia/Reperfusion by DUSP-Mediated Regulation of the ERK1/2/JNK Pathway

**DOI:** 10.1371/journal.pone.0102292

**Published:** 2014-07-14

**Authors:** Tongda Xu, Xin Wu, Qiuping Chen, Shasha Zhu, Yang Liu, Defeng Pan, Xiaohu Chen, Dongye Li

**Affiliations:** 1 Research Institute of Cardiovascular Diseases, Xuzhou Medical College, Xuzhou, Jiangsu, China; 2 The First Clinical College, Nanjing Traditional Chinese Medicine University, Nanjing, Jiangsu, China; Virginia Commonwealth University, United States of America

## Abstract

The purpose of this study was to observe the effects of salvianolic acid A (SAA) pretreatment on the myocardium during ischemia/reperfusion (I/R) and to illuminate the interrelationships among dual specificity protein phosphatase (DUSP) 2/4/16, ERK1/2 and JNK pathways during myocardial I/R, with the ultimate goal of elucidating how SAA exerts cardioprotection against I/R injury (IRI). Wistar rats were divided into the following six groups: control group (CON), I/R group, SAA+I/R group, ERK1/2 inhibitor PD098059+I/R group (PD+I/R), PD+SAA+I/R group, and JNK inhibitor SP600125+I/R group (SP+I/R). The cardioprotective effects of SAA on the myocardium during I/R were investigated with a Langendorff device. Heart rate (HR), left ventricular systolic pressure (LVSP), left ventricular end-diastolic pressure (LVEDP), maximum rate of ventricular pressure rise and fall (±dp/dt_max_), myocardial infarction areas (MIA), lactate dehydrogenase (LDH), and cardiomyocytes apoptosis were monitored. To determine the crosstalk betwee JNK and ERK1/2 via DUSP2/4/16 with SAA pretreatment, siRNA-DUSP2/4/16 were performed. The expression levels of Bcl-2, Bax, caspase 3, p-JNK, p-ERK1/2 and DUSP2/4/16 in cardiomyocytes were assayed by Western blot. Our results showed that LDH, MIA and cell apoptosis were decreased, and various parameters of heart function were improved by SAA pretreatment and SP application. In the I/R group, the expression levels of p-ERK1/2 and DUSP4/16 were not significantly different compared with the CON group, however, the protein expression levels of p-ERK1/2, Bcl-2 and DUSP4/16 were higher, while p-JNK, Bax, caspase 3 and DUSP2 levels were reduced among the SAA+I/R, PD+SAA+I/R and SP+I/R groups. The above indices were not significantly different between the SAA+I/R and SP+I/R groups. Compared with the SAA+I/R group, p-ERK1/2 was increased and p-JNK was decreased in the SAA+si-DUSP2+I/R, however, p-ERK was downregulated and p-JNK was upregulated in SAA+si-DUSP4+I/R group. SAA exerts an anti-apoptotic role against myocardial IRI by inhibiting DUSP2-mediated JNK dephosphorylation and activating DUSP4/16-mediated ERK1/2 phosphorylation.

## Introduction

Ischemic heart disease remains one of the leading causes of death all over the world, and its global prevalence is continuously increasing. Myocardial ischemia results in a lack of myocardial oxygen supply, which can damage myocardial structure and heart function. It is necessary for damaged myocardium to restore the supply of oxygen and nutrients and to improve its functional recovery through myocardial reperfusion. In most cases, the damaged structure and heart function can be restored to its basal condition through ischemia/reperfusion (I/R), however, in some cases reperfusion can augment ischemic injury of the heart, a situation termed as myocardial ischemia-reperfusion injury (IRI) [1].

A large body of literature indicates that cell apoptosis can be induced during I/R, and this is one of the main components involved in myocardial IRI. To date, some experiments and clinical studies have suggested that cell apoptosis may be an important link during the pathogenesis of myocardial IRI [2], [3].

The MAPK signaling pathway is believed to regulate the apoptosis of cardiomyocytes. The MAPKs are serine/threonine protein kinases activated by the phosphorylation of both threonine and tyrosine residues. The kinase family has three members in classical pathway, including extracellular signal-regulated kinases (ERK1/2), C-jun N-terminal kinase (JNK) and the protein kinase p38. Previous studies indicate that activated ERK1/2 contributes to cardioprotection against IRI via anti-apoptotic mechanisms, while the activation of JNK has the opposite effect and the effect of p38 on cell apoptosis for IRI myocardium remains controversial [4], [5].

In recent years, emerging research has been shown to possess a remarkable ability to deal with IRI. Currently, the correlative treatment strategies for myocardial IRI are mainly concentrated on ischemia preconditioning, drug and gene pretreatment and post-treatment. Drug pretreatment, especially pretreatment with Chinese medicines, has received increasing attention as a means of providing cardioprotection against myocardial IRI, with a large body of evidence demonstrating that traditional Chinese medicines rich in salvianolic acid exert effective protection for the myocardium against IRI [6], [7].

**Figure 1:**
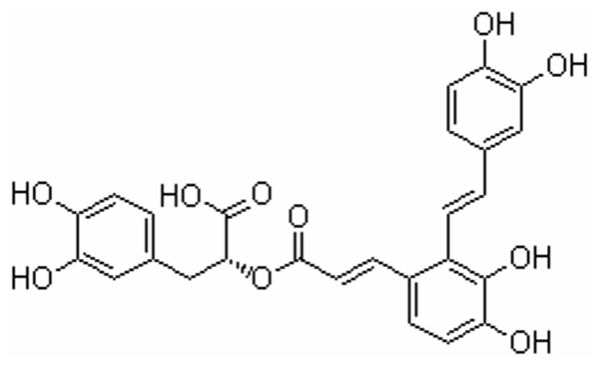
Chemical structure of SAA.

Salvianolic acids include salvianolic acid A (SAA), salvianolic acid B (SAB), rosmarinic acid and other polyphenolic acids. SAA ((2R)-3-(3, 4-dihydroxyphenyl)-2-[(E)-3-[2-[(E)-2-(3, 4-dihydroxyphenyl) ethenyl]-3, 4-dihydroxyphenyl] prop-2-enoyl] oxypropanoic acid, see Figure 1) is the main active constituent of *Salvia miltiorrhiza*.

SAA, a water-soluble component, is extracted from the dried root and rhizome of *Salvia miltiorrhiza* Bunge (Danshen), which possesses antioxidant, anti-inflammatory, antiplatelet properties. Recently, it has been suggested that SAA displays cardioprotective effects against myocardial IRI [6], [7], [8]. In spite of a large body of evidence showing protective effects of SAA on the myocardium during I/R, its role and cardioprotective mechanisms have not been clearly elucidated with respect to the apoptosis pathway [9], [10].

Fan et al [11] found that SAA pretreatment can further increase the protein expression of Bcl-2 and ERK1/2 in I/R cardiomyocytes. The establishment of models for I/R myocardial tissue in vivo and cardiomyocytes in vitro induced by H_2_O_2_ (H9c2) indicate that SAA may be involved in preventing cardiomyocytes apoptosis during I/R via ERK1/2 signaling. However, it is still unclear how this agent exerts its anti-apoptotic effects on I/R cardiomyocytes by modulating signaling through the ERK1/2 and JNK pathways, and concrete mechanisms are still lacking.

In order to elucidate the role and mechanism by which SAA pretreatment exerts cardioprotection against myocardial IRI, the present study was designed to evaluate the cardioprotective effects of SAA on IRI myocardium and attempted to discuss its mechanism, with the ultimate goal of establishing a theoretical and experimental basis for the use of SAA in the prevention and treatment of myocardial IRI.

## Materials and Methods

### Animals and materials

Approval for these experiments was obtained in advance from the Animal Ethics Committee of nanjing traditional chinese medicine university (permit number CMCACUC2009-04-135). Male Wistar rats (clean grade, Xuzhou Medical College, China) weighing 220–250 g were divided into the following groups: control group (CON, n = 6), I/R group (I/R, n = 6), SAA pretreatment group (SAA+I/R, n = 6), ERK1/2 inhibitor PD098059+I/R group (PD+I/R, n = 6), ERK1/2 inhibitor PD098059+SAA+I/R group (PD+SAA+I/R, n = 6) and JNK inhibitor SP600125+I/R group (SP+I/R, n = 6). SAA lyophilized powder (product number 20120203, purity >98%) was manufactured by Qing Feng Pharmaceutical Products (Jiangxi, China). SAA was dissolved in distilled water to a final concentration of 0.1 mM and these solutions were stored at 4°C for use as soon as possible. PD and SP were purchased from Cell Signaling Technology Inc (MA, USA).

### Isolated heart perfusion protocol

Rats were anesthetized with 10% chloral hydrate and their hearts were isolated and placed into a container of chilled Krebs–Henseleit buffer (KHB). KHB was composed of the following (in mM): 25.2 NaHCO_3_, 118 NaCl, 1.2 MgSO_4_, 1.2 KH_2_PO_4_, 4.7 KCl, 1.7 CaCl_2_ and 11.1 glucose. This buffer was bubbled with 95% O_2_/5% CO_2_ at 37°C and kept at a pH of 7.4. Retrograde perfusion of the hearts was performed in a non-recirculating Langendorff apparatus with the pressure being kept constant at 70 mm Hg. A latex balloon was placed in the left ventricle via left atrium and connected to a computer coupled with a bio-signal system (Taimeng Co, Chengdu, China) through a pressure transducer. All hearts were equilibrated for 30 min. Control group hearts were perfused with KHB for 150 min without ischemia. I/R group hearts were subjected to global ischemia for 30 min following reperfusion for 120 min with KHB solution. SAA+I/R group hearts were perfused with SAA (20 µM) for 30 min before I/R as described above [9]. For the PD+I/R group, hearts were pretreated with PD (20 µM) for 30 min following I/R as described above. PD + SAA+IR group hearts were initially perfused with 20 µM PD for 30 min, followed by the same procedure performed in the SAA+I/R group. In the SP+I/R group, SP (10 µM) pretreatment for 30 min was carried out prior to ischemia and reperfusion (120 min) as mentioned above. SAA and inhibitors were infused into the heart via a side pipe located just proximal to the heart cannula. The concentration of SAA (20 µM) was selected on the basis of preliminary experiments [9]. Left ventricular systolic pressure (LVSP), left ventricular end-diastolic pressure (LVEDP), maximum rate of ventricular pressure rise and fall (±dp/dtmax) and heart rate (HR) were recorded every 10 min during perfusion after equilibration.

### Determination of myocardium infarction areas (MIA)

MIA was assessed by triphenyltetrazolium chloride (TTC, Sigma-Aldrich, USA) staining as described previously [12]. Briefly, after the ventricular tissue reperfusion was finished, the tissue was sliced into 1-mm sections and incubated in 1% triphenyltetrazolium chloride for 15 min at 37°C. Subsequently, the sections were placed in a saline solution containing 10% formaldehyde for 1 h prior to removing the infracted (white) tissue, which was weighed in total. The weight of the infarction tissue was expressed as a percentage of the total ventricle weight.

### Measurement of lactate dehydrogenase (LDH) in coronary effluent

After 15 min reperfusion, the coronary effluent of each group was collected for LDH assay. LDH was assayed with the use of a commercially available assay kit according to the manufacturer's instructions (Jiancheng Bioengineering Institute, China). Analysis of all assayed samples was repeated three times.

### Evaluation of cell apoptosis

The terminal deoxynucleotidyl transferase-mediated biotinylated UTP nick end labeling (TUNEL) assay was performed using an in situ cell death detection kit (Roche, Swiss). The cardiomyocytes were rinsed twice in PBS again and then apoptotic cells were detected by TUNEL staining following the manufacturer's instructions. At least three heart tissues were chosen from each group. One hundred cells were counted in every viewed field for all 10 fields (cells were examined at ×400 magnification). Cardiomyocytes were stained with 4', 6-diamidino-2-phenylindole (DAPI) for staining all nuclei of cardiomyocytes, with the TUNEL method, only the nuclei of apoptotic cells stained brown, while normal nuclei stain blue with DAPI, and the ratio of TUNEL-positive cardiomyocytes was calculated as follows: (number of apoptotic cells/total number counted) ×100%. Each assay was performed in a blinded manner and the experiment was repeated three times.

### Isolation and culture of adult rat ventricular cardiomyocytes

Left ventricular cardiomyocytes were isolated from adult Wistar rats and cultured as described previously [12]. In short, isolated hearts were perfused for 5 min with Ca^2+^-free buffer. The hearts were then switched to the same perfusion buffer. The perfusate was recirculated at a flow rate of 6–10 ml/min. After a 25 min recirculation period, the hearts were removed from the cannula and the left ventricle tissues were cut into small pieces in Krebs–Bicarbonate (KB) solution (pH 7.2). Cardiomyocytes were harvested and filtered through 200µ meshes of nylon. The cells were then resuspended in pre-oxygenated KB solution and washed three times to remove dead cardiomyocytes. After isolation, 81%–87% of the viable cardiomyocytes were quiescent. Then, cells were cultured in Dulbecco's minimal essential medium (DMEM) containing 1% penicillin–streptomycin at a density of 2×10^4^ in a 12-well culture dish.

### Simulated I/R protocol for cardiomyocytes and DUSP transfection

Simulated I/R was performed as described previously [9], [12]. In CON group, cardiomyocytes were cultivated for 18 h. I/R group cardiomyocytes were cultivated for 13 h, and then placed in three gas incubator to simulate ischemia for 3 h. After this step, cardiomyocytes were cultured in high-glucose DMEM medium and kept in a CO_2_ incubator to simulate reperfusion for different lengths of time (0.5 h, 1 h, 2 h, 4 h) to determinate the optimal reperfusion time. Our preliminary experiment had determinated that reperfusion for 2 h was chosen as the optimal time point for further experiments in this study (concrete results were shown in “Results” section). In the SAA+I/R group, cardiomyocytes were cultivated for 1 h, and I/R was performed after pretreatment with 10 µM for 12 h. The optimal concentration of SAA was determined based on previous experiment [9]. In the PD+SAA+I/R group, cardiomyocytes were pretreated with PD for 30 min prior to SAA pretreatment, after incubation with SAA, I/R was performed. In the PD+I/R and SP+I/R groups, cardiomyocytes were pretreated with PD or SP for 30 min prior to ischemia, after ischemia, reperfusion was followed. Cardiomyocytes were transfected with the indicated vectors (siRNA-DUSP2/4/16) using the transfection reagents Lipofectamine 2000 (Invitrogen, USA) according to the manufacturer's instructions 48 h prior to induction of I/R, achieving approximately 60% transfection efficiency, then I/R was performed. To determine the effects of siRNA-DUSP2/4/16 and SAA on p-ERK and p-JNK, cardiomyocytes was divided into four group: 1. I/R group. 2. siRNA-DUSP+I/R group (si-DUSP+ I/R): cardiomyocytes was transfected siRNA-DUSP2/4/16 for 48 h prior to induction of I/R. 3. SAA+I/R group. 4. SAA+siRNA-DUSP+ I/R group (SAA+si-DUSP+ I/R): cardiomyocytes was transfected siRNA-DUSP2/4/16, then SAA pretreatment for 30 min before I/R.

### Measurement of the shortening amplitude of cardiomyocytes

After each group of cells (except those in the CON group) completed the reperfusion phase, a few drops of medium containing ventricular cardiomyocytes were added to an open chamber on the stage of an inverted microscope (Olympus, Japan). After the cells spontaneously attached to the bottom of the chamber, cardiomyocytes were superfused at 2 ml/min with KH buffer (containing 2.0 mM Ca^2+^ and 100 nM isoprenaline) at 37°C and adjusted to a pH 7.4 by equilibration (with a 95% O_2_ and 5% CO_2_ atmosphere). Isoprenaline increased the shortening amplitude of cardiomyocytes in a concentration-dependent manner, with 0.1 M isoprenaline exerting the maximal effect. Some rod-shaped ventricular cardiomyocytes with clear sarcomeres were chosen to undergo electrical stimulation at 0.5 Hz. At least 10 cardiomyocytes per heart from each group were evaluated. The whole procedure was recorded with a video recorder (Panasonic, Japan), and the output of the video edge detector was sent to a computer [9]. Ventricular myocardial contraction was indexed by the percent reduction in resting cell length following stimulation.

### Western blot analysis

After cardiomyocytes were cultured with or without SAA pretreatment, the cells were harvested and homogenized in lysis buffer containing proteinase inhibitor. The protein concentration in each sample was determined using a BCA protein assay kit (Bio-Rad, CA, USA). For immunoblotting, 40 µg of protein was separated by 15% SDS-polyacrylamide gel electrophoresis (PAGE) and subsequently transferred to a polyvinylidene difluoride (PVDF) membrane. Adequate transfer of protein was confirmed by Coomassie Blue staining of the gel and Ponceau Red staining of the membranes. Equal protein loading was confirmed by probing for β-actin, and the membranes were probed overnight at 4°C with rabbit polyclonal primary antibodies or mouse monoclonal antibodies (at a dilution of 1∶1000) against the following proteins: ERK1/2, JNK, dual specificity protein phosphatase 2 (DUSP2), dual specificity protein phosphatase 4 (DUSP4), dual specificity protein phosphatase 16 (DUSP16), phospho-ERK1/2 (p-ERK1/2), phospho-JNK (p-JNK), phospho-DUSP2 (p-DUSP2), phospho-DUSP4 (p-DUSP4), phospho-DUSP16 (p-DUSP16) (1∶1000; Cell Signaling Technology, MA, USA), Bcl-2, Bax, caspase 3 (1∶500; Santa Cruz, USA) and β-actin (1∶1000; Zhongshan, Beijing, China). The membranes were then incubated with anti-rabbit IgG or anti-mouse IgG secondary antibodies (1∶2000; Zhongshan, Beijing, China) for 2 h. Protein bands were visualized by nitro blue tetrazolium and 5-bromo-4-chloro-3-indolyl-phosphate. The membranes were scanned and the relative intensity of the bands was determined with the Image J 3.0 system. The optical density of the control group bands was set at 1 arbitrary densitometry unit.

### Statistical analyses

For each experimental series, data were presented as means±S.E.M. Statistical analysis was performed with GraphPad Prism 4.0 software. Statistical significance (P<0.05) for each variable was estimated by 1-way or 2-way analysis of variance followed by Bonferroni post-hoc tests.

## Results

### Hemodynamic effects of SAA on I/R heart in vitro

We performed an in vitro analysis of cardiac function following I/R. Compared with the CON group, the values of HR, LVSP and ±dp/dt_max_ were lower (P<0.05) and the level of LVEDP was increased (P<0.05) in the I/R, SAA+I/R, PD+I/R, PD+SAA+I/R and SP+I/R groups. Compared with the I/R group, improvements were seen in the HR, LVSP, ±dp/dt_max_ values of the SAA+I/R, PD+SAA+I/R and SP+I/R groups, and LVEDP could be lowered (P<0.05), however, HR, LVSP, LVEDP and ±dp/dt_max_ values were not significantly different in the PD+I/R group. Compared with the SAA+I/R group, HR, LVSP, ±dp/dt_max_ were decreased (P<0.05) and LVEDP was increased (P<0.05) in the PD+SAA+I/R group. We did not detect significant differences between the SP+I/R and SAA+I/R groups with regards to HR, LVSP, LVEDP and ±dp/dt_max_ (P>0.05). Compared with the PD+SAA+I/R group, HR, LVSP, ±dp/dt_max_ values were significantly increased (P<0.01), while LVEDP showed an obvious decrease (P<0.01) in SP+I/R group (see Table 1).

**Table 1:** Each group of Myocardial Function in Isolated Ischemia/Reperfused hearts.

		CON	I/R	PD+I/R	SAA+I/R	PD+SAA+I/R	SP+I/R
HR(Beats/min)	**a**	249.60±6.83	239.40±9.96	235.60±5.94	240.60±4.22	243.60±4.33	244.00±4.57
	b	246.00±6.54	179.60±4.06**	180.20±6.41**	211.00±4.64* $	185.60±10.19* #	203.80±9.92* ^$&^
	c	240.00±6.04	174.200±4.47**	175.80±5.86**	204.60±4.29* $	180.40±9.66* #	198.20±9.81* ^$&^
LVSP(mmHg)	a	126.60±4.57	113.60±5.27	115.60±5.20	122.00±4.12	120.60±4.17	123.80±4.44
	b	122.20±3.72	73.40±1.75**	77.80±2.25**	106.60±2.94* $	90.00±5.48* ^$#^	100.40±2.38* ^$&^
	c	116.00±4.38	69.60±1.75**	72.80±2.01**	100.8±2.20* $	84.60±4.80* ^$#^	93.60±52.29* ^$&^
**LVEDP(mmHg)**	a	11.26±1.21	13.46±0.87	12.92±0.75	11.34±0.89	12.34±0.74	11.42±0.67
	b	12.40±0.76	22.40±1.97**	21.80±1.39**	16.80±0.95* $	20.900±0.929* ^$#^	17.38±0.28** ^$&^
	c	13.24±0.60	22.88±2.03**	22.18±1.41**	17.280±1.011* $	21.40±0.98* ^$#^	17.82±0.28* ^$&^
+dp/dt**(mmHg/s)**	a	2496.60±48.92	2451.60±27.84	2471.00±20.50	2528.60±20.33	2579.20±13.79	2561.80±38.60
	b	2450.60±45.52	1328.40±72.91**	1379.60±67.18**	1828.40±49.51** $	1525.20±31.03** ^$#^	1770.80±49.28** ^$&^
	c	2415.40±32.50	1246.60±94.98**	1281.20±53.04**	1653.00±59.30** $	1453.60±35.54** ^$#^	1658.60±49.39** ^$&^
-dp/dt**(mmHg/s)**	a	1983.00±51.30	1980.60±52.43	2007.20±45.26	1985.80±23.75	2009.40±29.34	1982.80±26.68
	b	1925.80±44.25	1455.80±38.12**	1503.20±52.04**	1742.20±36.23* $	1554.00±25.81* ^$#^	1677.60±33.04* ^$&^
	c	1864.20±47.72	1435.60±37.00**	1490.20±52.06**	1725.60±36.91* $	1542.80±26.85* ^$#^	1671.20±34.14* ^$&^

a. Baseline. b. Perfusion 30 min. c. Perfusion 120 min.

Retrograde perfusion of the hearts was performed in a non-recirculating Langendorff apparatus with. A latex balloon was placed in the left ventricle via left atrium and connected to a computer coupled through a pressure transducer. The concentration of SAA (20 µM), PD (20 µM), SP (10 µM) used were chosen to performed the experiments. SAA+I/R group hearts were perfused with SAA for 30 min before I/R, for the PD+I/R group, hearts were pretreated with PD for 30 min following I/R, PD + SAA+IR group hearts were perfused with PD for 30 min, followed by the same procedure performed in the SAA+I/R group.

*P<0.05.

**P<0.01 versus CON group.

$ P<0.05.

$$ P<0.01 versus I/R

# P<0.05

## P<0.01 versus SAA+I/R

& P<0.05

&& P<0.01 versus PD+SAA+I/R.

All data were expressed as mean ±SEM, n = 6.

### Effects of SAA on MIA of I/R heart in vitro

**Figure 2:**
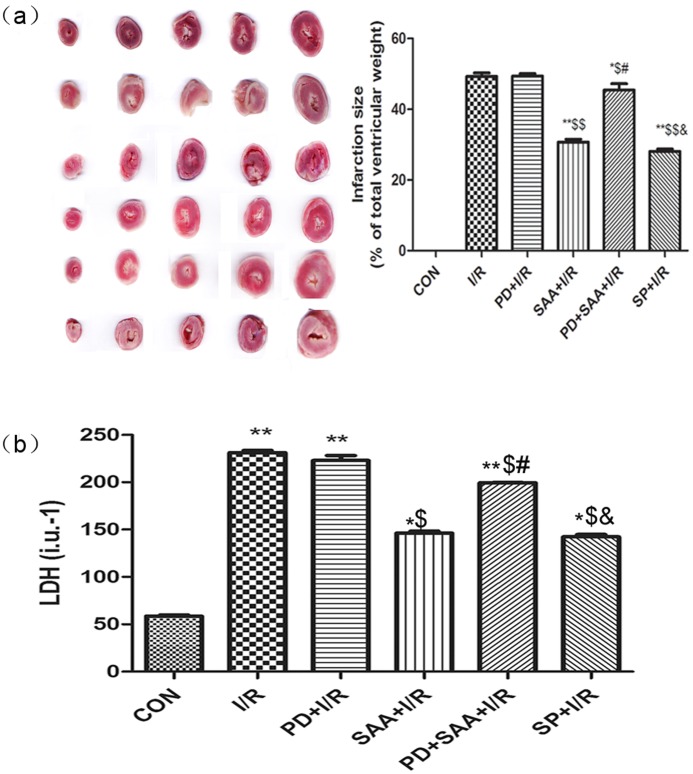
Effects of SAA on MIA and LDH of coronary effluent. (**a**) Effects of SAA and SP600125 on MIA. After the ventricular tissue reperfusion was finished, the tissue was sliced into 1-mm sections. The weight of the infarction tissue was expressed as a percentage of the total ventricle weight. (**b**) Effects of SAA and SP600125 on LDH of coronary effluent. After 15-min reperfusion, the coronary effluent of each group was collected for LDH assay. *P<0.05, **P<0.01 versus CON group, ^$^P<0.05, ^$$^P<0.01 versus I/R, ^#^P<0.05, ^##^P<0.01 versus SAA+I/R, ^&^P<0.05, ^&&^P<0.01 versus PD+SAA+I/R. All data were expressed as mean ±SEM, n = 6.

Compared with the CON group, MIA were increased following I/R (49.29±1.000% vs 0.00±0.00, P<0.05); however, this parameter could be reduced by pretreatment with SAA (P<0.05). Compared with the I/R group, MIA in the SAA+I/R, PD+SAA+I/R and SP+I/R groups was diminished (30.72±0.79, 45.42±1.81, 28.06±0.65% vs 49.29±1.00%, P<0.05). However, this value in the PD+I/R group did not yield any significant effect on MIA compared with the I/R group (49.36±0.73% vs 49.29±1.00%, P>0.05). Compared with the PD+I/R group, the results were equivalent to I/R group comparing with the SAA+I/R, PD+SAA+I/R and SP+I/R groups. MIA were increased in the PD+SAA+I/R group relative to the SAA+I/R group (P<0.05). However, MIA was not found to be significantly different between the SP+I/R and SAA+I/R groups (28.06±0.65% vs 30.72±0.79%, P>0.05). Further, compared with the PD+SAA+I/R group, MIA were obviously decreased in the SP+I/R group (28.06±0.65 vs 45.42±1.81, P<0.05) (see Figure 2a).

### Effects of SAA on LDH of coronary effluent

Compared with the CON group, LDH values was increased following I/R (231.40±2.31 vs 58.40±1.12, P<0.01). Further, compared with the I/R group, LDH values was lower in the SAA+I/R, PD+SAA+I/R and SP+I/R groups (146.40±2.07, 208.30±4.51, 142.30±2.55 vs 231.40±2.31, P<0.05), however, this value in the PD+I/R group did not yield any significant effect on LDH compared with the I/R group (220.23±2.14% vs 231.40±2.31%, P>0.05). Compared with the PD+I/R group, the results were equivalent to I/R group comparing with the SAA+I/R, PD+SAA+I/R and SP+I/R groups. LDH was increased in the PD+SAA+I/R group relative to the SAA+I/R group (208.30±4.51 vs 146.40±2.07, P<0.05); however, LDH values were not found to be significantly different between the SAA+I/R and SP+I/R groups (P>0.05). Further, compared with the PD+SAA+I/R group, LDH values were obviously decreased in the SP+I/R group (208.30±4.51 vs 142.30±2.55, P<0.05) (see Figure 2b).

### Effects of SAA on apoptosis of I/R-injured myocardium in vitro

**Figure 3:**
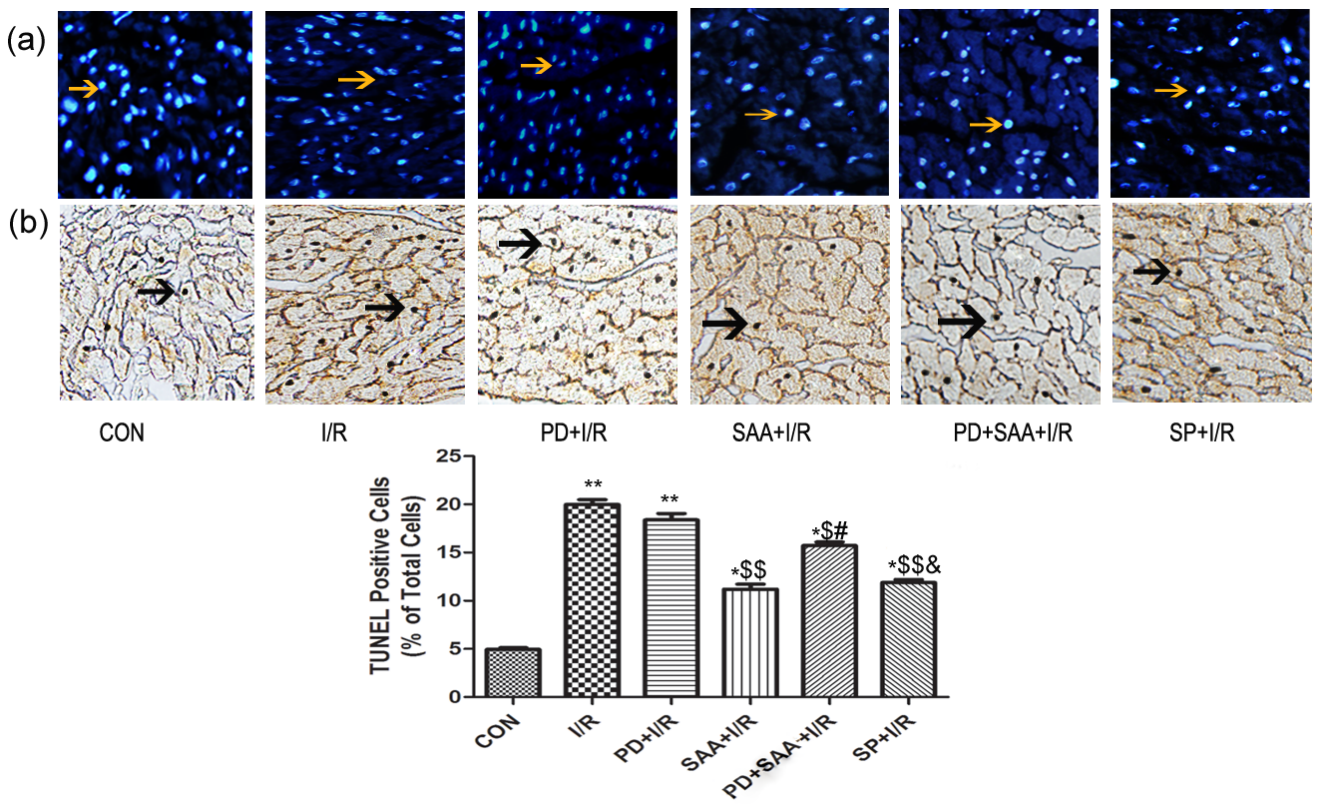
Effects of SAA on apoptosis of I/R myocardium in vitro. A representative photomicrograph of DAPI-stained (Figure 3a) and TUNEL (Figure 3b) cardiomyocytes were showed. After 2 h reperfusion, the heart tissure were sectioned for analysis of anti-apoptotic effect of SAA, PD and SP, cardiomyocytes were stained with DAPI, and the ratio of TUNEL-positive cardiomyocytes was calculated. *P<0.05, **P<0.01 versus CON group, ^$^P<0.05, ^$$^P<0.01 versus I/R, ^#^P<0.05, ^##^P<0.01 versus SAA+I/R, ^&^P<0.05, ^&&^P<0.01 versus PD+SAA+I/R. All data were expressed as mean ±SEM, n = 6. All data were expressed as mean ±SEM, n = 6. Cells were examined by light microscopy (200×magnification). Yellow allows indicate DAPI-stained nucleus, black allows indicate TUNELpositive caryons.

Compared with CON group, the rate of cardiomyocyte apoptosis increased following I/R (19.99±0.50% vs 4.96±0.15%, P<0.01). Compared with I/R group, the apoptosis rate was reduced in cardiomyocytes belonging to the SAA+I/R, PD+SAA+I/R and SP+I/R groups (11.19±0.52, 15.72±0.37, 11.89±0.29% vs 19.99±0.50%, P<0.05), however, this value in the PD+I/R group did not yield any significant effect on apoptosis compared with the I/R group (18.38±0.67% vs 19.99±0.50%, P>0.05). Compared with the PD+I/R group, the results were equivalent to I/R group comparing with the SAA+I/R, PD+SAA+I/R and SP+I/R groups. The rate of cardiomyocytes apoptosis was increased in the PD+SAA+I/R group relative to the SAA+I/R group (15.72±0.37% vs 11.19±0.52%, P<0.05); however, this parameter was not significantly different between the SAA+I/R and SP+I/R groups (P>0.05). Further, compared to the PD+SAA+I/R group, the apoptosis rate was markedly decreased in the SP+I/R group (15.72±0.37% vs 11.89±0.29%, P<0.05). These results indicate that when the ERK1/2 pathway was inhibited during the course of I/R, the myocardial hemodynamic parameter, LDH values, MIA and cell apoptosis are not significantly changed, as compared to the I/R group. Therefore, further tests of the PD+I/R cardiomyocytes were not performed (see Figure 3).

### Effect of SAA on single cardiomyocyte contractile function

**Figure 4:**
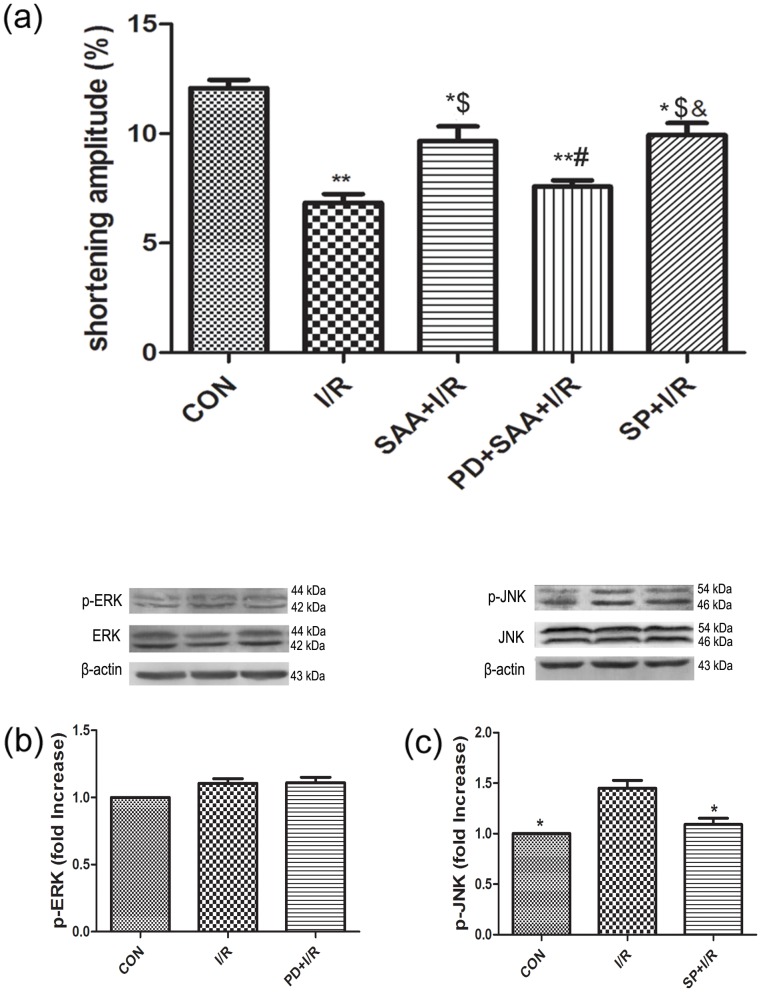
The effect of I/R on single cardiomyocytes contractile function and the expression of JNK, ERK during I/R. **(a) Effect of SAA on single cardiomyocytes contractile function**. After the cells spontaneously attached to the bottom of the chamber, cardiomyocytes were superfused. At least 10 cardiomyocytes per heart from each group were evaluated. Ventricular myocardial contraction was indexed by the percent reduction in resting cell length following stimulation. *P<0.05, **P<0.01 versus CON group, ^$^P<0.05, ^$$^P<0.01 versus I/R, ^#^P<0.05, ^##^P<0.01 versus SAA+I/R, ^&^P<0.05, ^&&^P<0.01 versus PD+SAA+I/R. **(b) The expression of p-ERK1/2(44 KDa, 42 KDa) during I/R.** *P<0.05 versus I/R, ^$^P<0.05 versus I/R. **(c) The expression of JNK(54 KDa, 46 KDa), p-JNK(54 KDa, 46 KDa) during I/R.** *P<0.05 versus I/R, ^$^P<0.05 versus I/R. Hearts were pretreated with PD (20 µM) or SP (10 µM)for 30 min following I/R. All data were expressed as mean ±SEM, n = 3.

Compared with the CON group, the shortening amplitude of single cardiomyocytes was decreased following I/R (6.84±0.40% vs 12.08±0.37%, P<0.01); however, this value was increased by pretreatment with SAA (9.66±0.67% vs 6.84±0.41%, P<0.05). Compared with the I/R group, the shortening amplitude of single cardiomyocytes in the SAA+I/R, PD+SAA+I/R and SP+I/R groups was increased (9.66±0.671, 7.59±0.29, 9.95±0.54% vs 6.84±0.40%, P<0.05). The shortening amplitude of single cardiomyocytes was decreased in the PD+SAA+I/R group relative to the SAA+I/R group (7.59±0.29% vs 9.66±0.67%, P<0.05). A significant difference was not observed between the SAA+I/R and SP+I/R groups with regards to the shortening amplitude of single cardiomyocytes (P>0.05). Additionally, compared with the PD+SAA+I/R group, the shortening amplitude of single cardiomyocytes was markedly increased in the SP+I/R group (9.95±0.54% vs 7.59±0.29%, P<0.01) (see Figure 4a).

### Activation of the JNK and ERK1/2 pathways during I/R

PD and SP were employed to further investigate the activation of ERK1/2 and JNK during I/R. The protein expression levels of total ERK1/2 and JNK were not significantly different among the CON, I/R, PD+I/R and SP+I/R groups (P>0.05). I/R was found to significantly activate p-JNK expression, as compared with the CON group (P<0.01); however, this trend could be partially reversed by adding SP(see Figure 4c). The protein expression level of p-ERK1/2 was not significantly different between the I/R and CON groups (see Figure 4b).

### The effects of different reperfusion time on p-ERK and p-JNK

**Figure 5:**
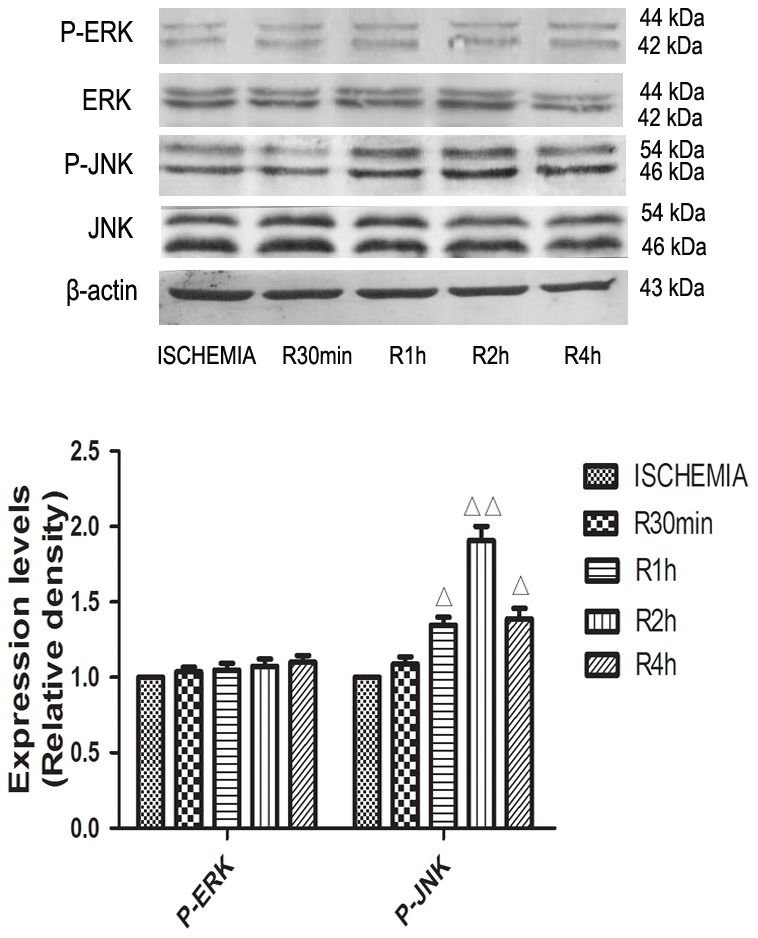
The effects of different reperfusion time on p-ERK and p-JNK. Cardiomyocytes were cultivated for 13(0.5 h, 1 h, 2 h, 4 h), the influence of different time of reperfusion on p-ERK(44 KDa, 42 KDa) and p-JNK(54 KDa, 46 KDa) was monitored. All data were expressed as mean ±SEM, n = 3, ^△^P<0.05, ^△△^P<0.01 versus ISCHEMIA group.

To determine the activated effect of different reperfusion time on p-ERK and p-JNK, the influence of different time of reperfusion (0, 0.5 h, 1 h, 2 h, 4 h) on p-ERK and p-JNK was monitored by western blot. Compared to the ischemia group, the expression level of p-JNK was elevated among different reperfusion groups (0.5 h, 1 h, 2 h, 4 h), however, the expression level of p-ERK had no significant difference from ischemia to different reperfusion groups. Among different reperfusion groups, the expression levels of p-JNK were step by step increased with reperfusion time prolonging and had significant differences (0.5 h, 1 h, 2 h), with peak level observed at reperfusion 2 h and reduced level at subsequent time points for 4 h. These results have demonstrated that the expression level of p-JNK reach the maximum value at reperfusion 2 h, therefore, reperfusion 2 h was chosen as the optimal reperfusion time for further experiments in this study(Figure5).

### Effects of SAA on ERK1/2, JNK, Bcl-2, Bax and caspase 3

The protein expression levels of total-ERK1/2 and total-JNK were not significantly different among all groups (P>0.05). Compared to the CON group, I/R had no effect on the expression of p-ERK1/2 (P>0.05) but downregulated the expression of Bcl-2 (P<0.05), however, the change in Bcl-2 levels could be partially reversed by SAA pretreatment (P<0.05). Compared with the I/R group, the protein expression levels of p-ERK1/2 and Bcl-2 were uprgulated in the SAA+I/R, PD+SAA+I/R and SP+I/R groups (P<0.05). Further, the protein expression levels of p-ERK1/2 and Bcl-2 were attenuated in the PD+SAA+I/R group relative to the SAA+I/R group (P<0.05). Significant differences in the expression levels of ERK1/2 and Bcl-2 were not observed between the SAA+I/R and SP+I/R groups (P>0.05). And compared with the PD+SAA+I/R group, there were marked high in the protein expression levels of p-ERK1/2 and Bcl-2 in SP+I/R group (P<0.01).

**Figure 6:**
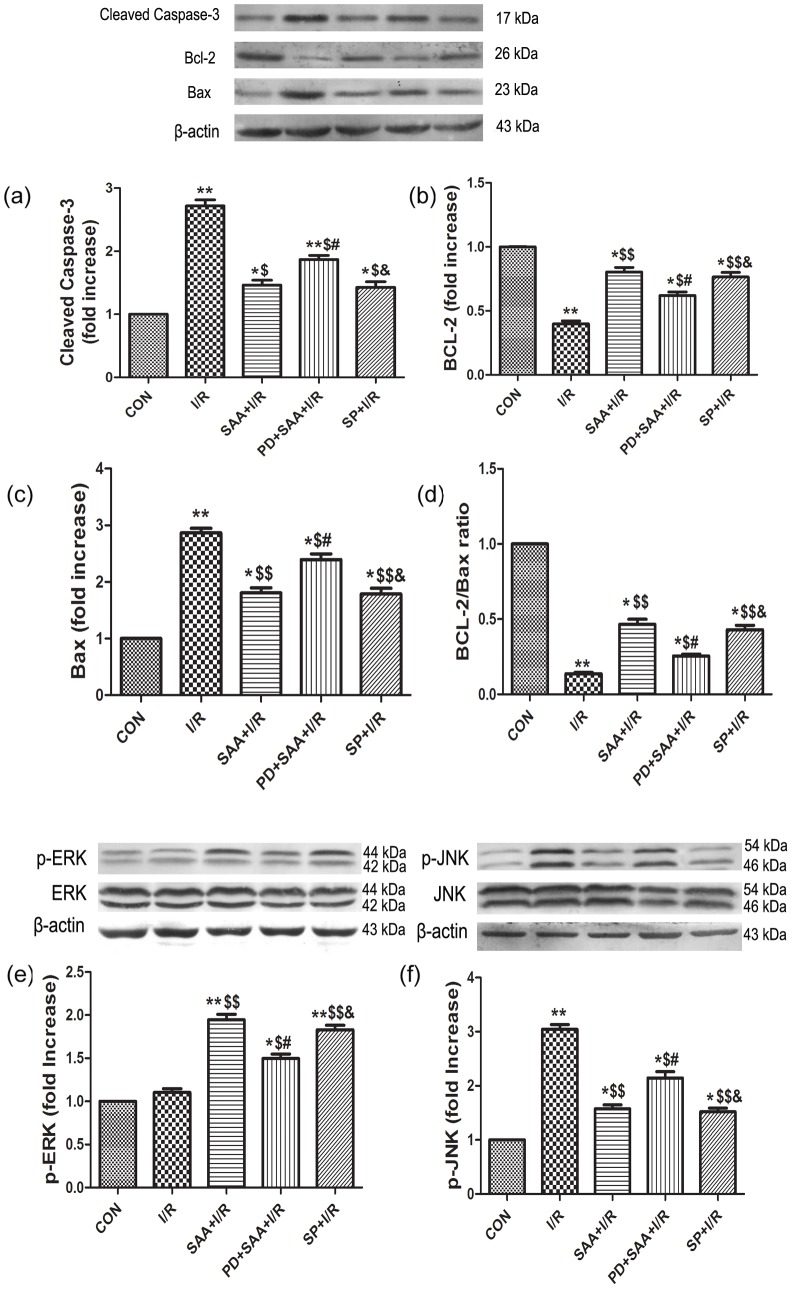
The effects of SAA and SP600125 on the expression of Bcl-2, Bax,caspase 3, Bcl-2/Bax, p-ERK1/2, p-JNK. a: caspase 3, b: Bcl-2, c: Bax, d: Bcl-2/Bax, e: p-ERK1/2(44 KDa, 42 KDa), f: p-JNK(54 KDa, 46 KDa). After 2 h reperfusion, the myocytes were harvested to detect protein expressions by western blot analysis. All data were expressed as mean ±SEM, n = 3, *P<0.05, **P<0.01 versus CON group, ^$^P<0.05, ^$$^P<0.01 versus I/R, ^#^P<0.05, ^##^P<0.01 versus SAA+I/R, ^&^P<0.05, ^&&^P<0.01 versus PD+SAA+I/R.

Comparisons of the I/R and CON groups revealed that the protein expression levels of p-JNK, Bax and caspase 3 were higher following I/R (P<0.05), however, this effect could be reversed by pretreatment with SAA (P<0.05). Compared with the I/R group, p-JNK, Bax and caspase 3 expression levels in the SAA+I/R, PD+SAA+I/R and SP+I/R groups were lowered (P<0.05). The protein expression levels of p-JNK, Bax and caspase 3 were increased in the PD+SAA+I/R group relative to the SAA+I/R group (P<0.05). Significant differences were not observed between the SAA+I/R and SP+I/R groups (P>0.05). Further, compared with the PD+SAA+I/R group, p-JNK, Bax and caspase 3 protein levels were decreased in the SP+I/R group (P<0.05) (see Figure 6).

### Effects of SAA on DUSP2, DUSP4 and DUSP16

Compared with the CON group, DUSP2 expression levels were increased following I/R (P<0.05), however, the increase could be reversed by pretreatment with SAA (P<0.05). Compared with the I/R group, DUSP2 expression was lower in the SAA+I/R, PD+SAA+I/R and SP+I/R groups (P<0.05). The protein expression level of DUSP2 was higher in the PD+SAA+I/R group relative to the SAA+I/R group (P<0.05), while levels were not significantly different between the SP+I/R and SAA+I/R groups (P>0.05). Further, compared with the PD+SAA+I/R group, DUSP2 protein expression was downregulated in the SP+I/R group (P<0.05).

**Figure 7:**
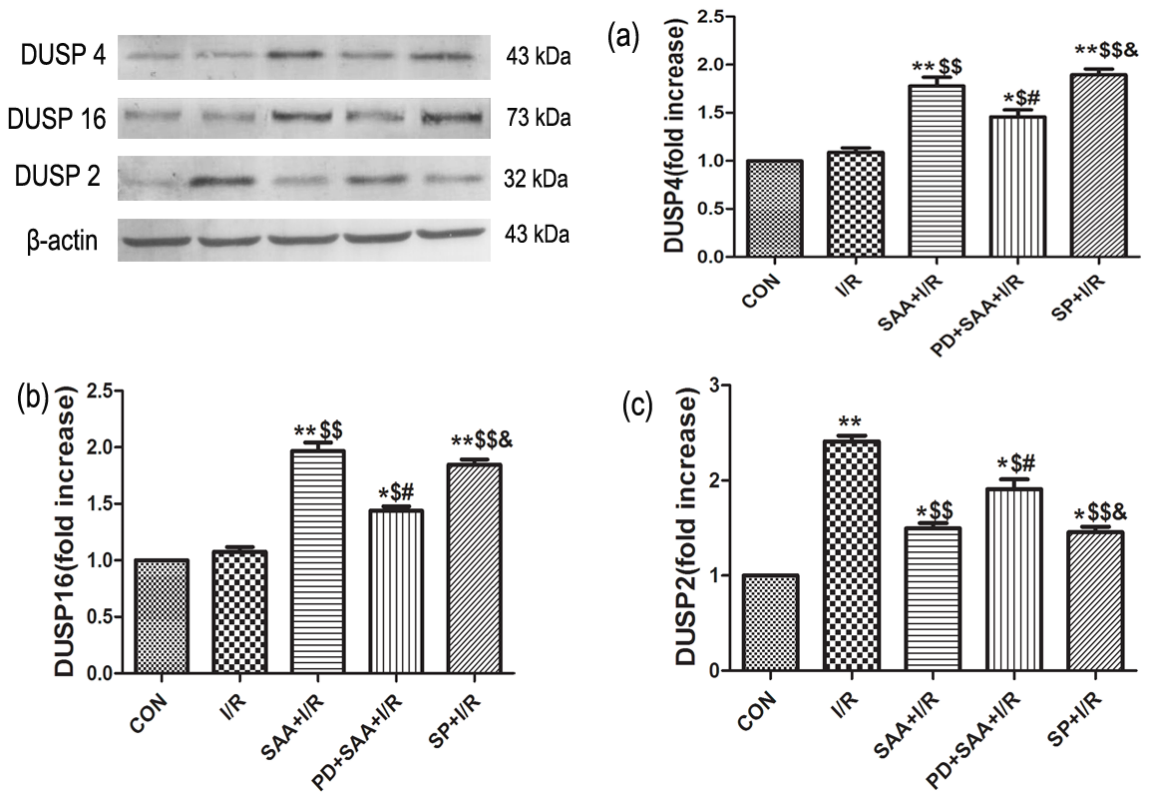
Effects of SAA on DUSP2, DUSP4, DUSP16 during I/R. In the SAA+I/R group, cardiomyocytes were cultivated for 1 h, and I/R was performed after pretreatment with 10 µM for 12 h. In the PD+SAA+I/R group, cardiomyocytes were pretreated with PD for 30 min prior to SAA pretreatment, after incubation with SAA, I/R was performed. In the SP+I/R groups, cardiomyocytes were pretreated with SP for 30 min prior to ischemia, after ischemia, reperfusion was followed. Bar graph a, b, c representative DUSP2, DUSP16, DUSP4 respectively. All data were expressed as mean ±SEM, n = 3, *P<0.05, **P<0.01 versus CON. ^$^P<0.05, ^$$^P<0.01versus I/R. ^#^P<0.05 versus SAA+I/R. ^&^P<0.05 versus PD+SAA+I/R.

The expression levels of DUSP4 and DUSP16 were not significantly altered during I/R by comparsion with the CON group (p>0.05), however, the values of DUSP4 and DUSP16 could be increased by SAA pretreatment (P<0.05). Compared with the I/R group, the protein expression levels of DUSP4 and DUSP16 in the SAA+I/R, PD+SAA+I/R and SP+I/R groups were elevated (P<0.05). The protein expression levels of DUSP4and DUSP16 were also lower in the PD+SAA+I/R group relative to the SAA+I/R group (P<0.05). There were no significant differences between the SAA+I/R and SP+I/R groups with regards to the protein expression of DUSP4 and DUSP16 (P>0.05). Further, compared with the PD+SAA+I/R group, DUSP4 and DUSP16 protein levels were higher in the SP+I/R group (P<0.05) (see Figure 7).

### The effects of SAA and siRNA-DUSP4/16 on p-ERK and p-JNK

**Figure 8:**
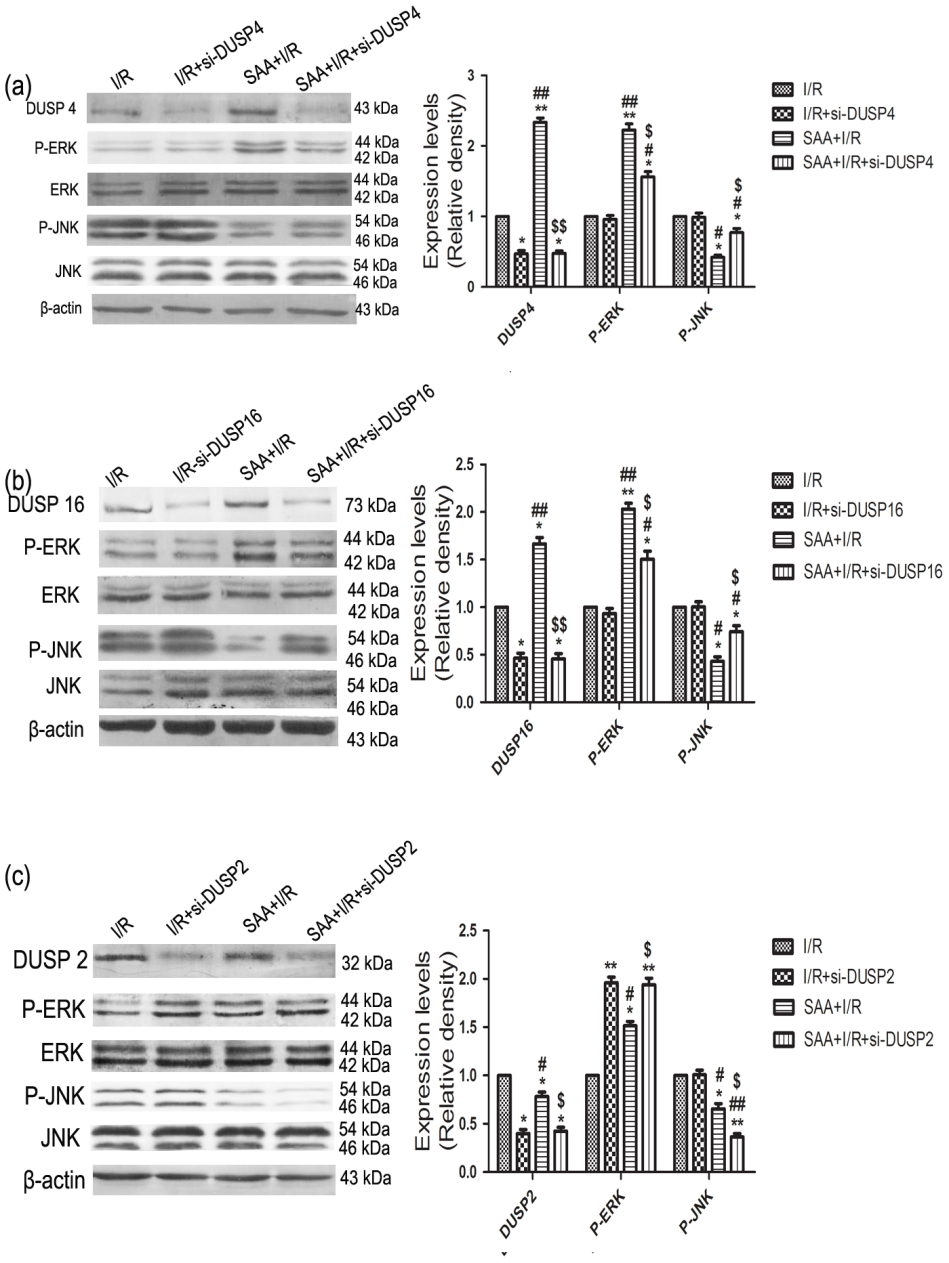
The effects of SAA and siRNA-DUSP2/4/16 on p-ERK and p-JNK. (a) Effects of SAA and siRNA-DUSP4 on p-ERK(44 KDa, 42 KDa) and p-JNK(54 KDa, 46 KDa); (b) Effects of SAA and siRNA-DUSP16 on p-ERK and p-JNK; (C) Effects of SAA and siRNA-DUSP2 on p-ERK and p-JNK; Cardiomyocytes was transfected siRNA-DUSP2/4/16, then SAA pretreatment for 30 min before I/R. All data were expressed as mean ±SEM, n = 3, *P<0.05, **P<0.01 versus I/R group. ^#^P<0.05,^ ##^P<0.01 versus si-DUSP+I/R group. ^$^P<0.05, ^$$^P<0.01versus SAA+ I/R group.

Compared with the I/R group, DUSP4 expression level was increased in SAA+I/R group (P<0.05) and decreased in si-DUSP4+I/R group (P<0.05). By comparing with the si-DUSP4+I/R group, there was no significant difference for DUSP4 expression level in SAA+si-DUSP4+I/R group (P>0.05), however, DUSP4 expression level was increased in SAA+I/R group relative to SAA+si-DUSP4+I/R group (P<0.05). Meanwhile, p-ERK expression levels had no significant difference between I/R group and si-DUSP4+I/R group (P>0.05), however, p-ERK expression levels were upregulated in SAA+I/R group and SAA+si-DUSP4+I/R group compared with I/R group and si-DUSP4+I/R group (P<0.05). Further, compared with the SAA+I/R group, p-ERK protein expression level was downregulated in the SAA+si-DUSP4+I/R group (P<0.05). As p-JNK, the expression levels had no significant difference between I/R group and si-DUSP4+I/R group(P>0.05), the change in p-JNK expression level could be attenuated by SAA pretreatment relative to I/R group (P<0.05), however, the p-JNK expression level was decreased in SAA+si-DUSP4+I/R group than those of I/R group and si-DUSP4+I/R group(P<0.05). Compared with the SAA+I/R group, the p-JNK expression level was marked high in SAA+si-DUSP4+I/R group (P<0.01) (Figure 8a).

The effects of siRNA-DUSP16 on p-ERK and p-JNK had the same results as those of siRNA-DUSP4 (The above-mentioned results) (Figure 8b).

### The effects of SAA and siRNA-DUSP2 on p-ERK and p-JNK

The protein expression levels of total-ERK1/2 and total-JNK were not significantly different among all groups (P>0.05). Compared to the I/R group, si-DUSP2+I/R had downregulated the expression of DUSP2 (P<0.05), however, the change in DUSP2 level could be partially reversed by SAA pretreatment (P<0.05). Compared with the SAA+I/R group, the protein expression level of DUSP2 was downregulated in the SAA+si-DUSP2+I/R (P<0.05). Further, the protein expression level of p-ERK1/2 was increased in the si-DUSP2+I/R group relative to the I/R group (P<0.05). Compared with the si-DUSP2+I/R group, the protein expression level of p-ERK1/2 was slightly reduced, but no significant difference in the SAA+si-DUSP2+I/R group, while p-ERK1/2 expression level was increased in the SAA+si-DUSP2+I/R compared with SAA+I/R group(P<0.05). The p-JNK expression level had no significant difference between I/R group and si-DUSP2 +I/R group(P>0.05), however, the p-JNK expression level was significantly decreased in SAA+I/R group and SAA+si-DUSP2+I/R group (P<0.01). Further, compared with the SAA+I/R group, p-JNK protein expression level was downregulated in the SAA+si-DUSP2+I/R group (P<0.05) (Figure 8c).

## Discussion

The impairment of left ventricular systolic and diastolic function, an initial pathophysiological change taking place during I/R, was reflected by hemodynamics and myocardial mechanics indices, including LVSP, LVEDP, ±dp/dt_max_ and HR. Of these parameters, LVSP, LVEDP and +dp/dt_max_ are indices of myocardial contractile ability [13]. HR can partly reflect the left ventricular contractive and diastolic function, and -dp/dt_max_ also serves as an indicator of left ventricular compliance [14]. As indicated by the results shown in Table 1, SAA pretreatment protects heart function following I/R. Further, these parameters showed no significant differences in the PD+I/R group, suggesting that the JNK pathway, and not the ERK1/2 pathway, was mainly activated during I/R.

LDH is one of specific enzymes present in myocardial cytoplasm, and its values can indirectly reflect the degree of myocardial IRI. In recent years, some studies have found that apoptosis may be one of the important steps in the pathogenesis of myocardial IRI, with the extent of cell apoptosis closely related to the severity of the myocardial IRI [11], [15]. In our experiments, SAA pretreatment significantly reduced MIA, LDH and cell apoptosis. However, there were no significant differences between the I/R and PD+I/R groups in these parameters. Further, these values were decreased in the PD+SAA+I/R and SP+I/R groups. This suggests that the ERK1/2 pathway is inhibited during I/R, and that SAA pretreatment induces ERK1/2 pathway activation to exert its cardioprotective effects.

Most studies suggest that members of the Bcl-2 family are key regulators of physiological and pathological apoptosis. This family consists of both cell death promoters, such as Bax and Bad, and cell death inhibitors, which include Bcl-2, Bcl-X and Mcl-1. It is well known that the activation of a family of aspartate-specific proteases termed caspases occurs in the execution phase of apoptosis. The cleavage of caspase-3 is often seen as the final step of the process that promotes the start of the apoptotic signaling pathway. In addition, the ratio of Bcl-2/Bax protein has also been suggested to determine survival or death after I/R [16], [17]. Our results indicated that during I/R, the expression level of Bcl-2 was downregulated while the expression levels of Bax, caspase 3 were upregulated. SAA pretreatment was able to inhibit cardiomyocyte apoptosis by upregulating the expression of Bcl-2 while also downregulating the expression levels of Bax, caspase 3, thereby increasing the Bcl-2/Bax ratio. These results indicate that SAA may exert its cardioprotective effect through the upregulation of Bcl-2 protein expression and downregulation of Bax, caspase 3 protein expression.

A large body of evidence has shown that SAA plays a vital cardioprotective role against IRI, particularly the apoptosis component of myocardial IRI [11], [18]; however, the cardioprotective role of SAA and the precise mechanism by which it exerts anti-apoptotic effects during myocardium I/R requires further clarification.

The results had shown that the expression of p-ERK1/2 was not significantly changed after I/R compared with CON group, while increased after pretreatment with SAA and decreased significantly in PD+SAA+I/R group. The above-mentioned results had shown ERK1/2 pathway could be activated with SAA pretreatment, however, the effect of SAA was reduced with adding PD, which suggested SAA could play a cardioprotection role through ERK1/2 pathway.

In our study, we demonstrate that the ERK1/2 signaling pathway is not activated during I/R, however, SAA pretreatment causes the ERK1/2 signaling pathway to be activated, upregulating p-ERK1/2 protein expression. SAA and SP have the similar cardioprotective effects during I/R from Fig 4b and 6e, it is worth considering that the anti-apoptotic effects of SAA in I/R cardiomyocytes are not just dependent on signaling through the ERK1/2 pathway, but may also rely on inhibition of the JNK pathway.

In order to further explore how SAA inhibits the JNK pathway to exert anti-apoptotic effects on I/R cardiomyocytes, SAA and JNK inhibitors were added during I/R. Our results indicate that the protein expression levels of p-JNK are downregulated by SAA pretreatment and JNK inhibitors, indicating SAA may exert anti-apoptotic effects on I/R cardiomyocytes by inhibition of JNK and upregulation of p-ERK1/2.

Some studies have shown that the ERK1/2 and JNK signaling pathways are not independent for each other, and increasing attention, both domestic and overseas, has been paid to crosstalk between the two pathways [19], [20]. Activated ERK1/2 and JNK access the nucleus by translocation to activate downstream substrates, regulate cell growth and affect cell function [21], [22].

ERK1/2 and JNK is regulated by upstream kinases (MAPK kinases) that activate each other in a step-by-step fashion. Transcription factors is the main targets of ERK1/2 and JNK, as ERK1/2 and JNK can phosphorylate a variety of transcription factors involved in transcription regulation of numerous genes [23], [24]. DUSP2, DUSP4 and DUSP16, as major target points of MAPK phosphatase, are involved in the regulation of ERK1/2 and JNK [25].

Current studies indicate that DUSP2, DUSP4 and DUSP16 are involved in the co-adjustment between ERK1/2 and JNK, with these reports focusing on tumor cells and immune response [26], [27], [28]. Katagiri et al [29] found that DUSP4 and DUSP16 can be activated by phosphorylation of ERK1/2-mediated Ser 446 in human cervical cancer cells and fibroblasts. In addition, ERK1/2 can dephosphorylate JNK and inhibit the activity of JNK by the activation of induced-DUSP4 in canine renal epithelial cells [30]. Meanwhile, DUSP2 is regarded as a potential mediator of the JNK-ERK1/2 crosstalk, and JNK can activate DUSP2 by positive modulation, which leads to further dephosphorylation of ERK1/2; thus, ERK1/2 protein expression was reduced and ERK1/2 activation was inhibited [31], [32], [33]. However, it is worth nothing that the above-mentioned studies were focused on tumor cells and not cardiomyocytes during I/R.

Our present results clearly demonstrate that the protein expression level of DUSP2 is higher during I/R. Further, the application of SAA and SP caused p-JNK and DUSP2 protein expression to be significantly downregulated, indicating that DUSP2 may mediate the regulation of ERK1/2 via JNK pathway.

In addition, we observed that the expression of DUSP4/16 was not significantly altered by I/R, however, the levels of p-ERK1/2 and DUSP4/16 were significantly upregulated, while p-JNK protein expression was downregulated by the application of SAA and SP. With the activation of ERK1/2, DUSP4/16 was upregulated while JNK was dephosphorylated and inhibited. Inhibition of ERK1/2 pathway resulted in the downregulation of DUSP4/16 and JNK was activated through negative feedback.

In Figure 8a and Figure 8b, p-ERK1/2 expression level was upregulated in SAA+siRNA-DUSP4/16 group compared with siRNA- DUSP4/16 group, which demonstrated DUSP4/16 maybe was downstream of ERK1/2 pathway, p-ERK1/2 expression was not inhibited plus siRNA-DUSP4/16 but increased with SAA pretreatment. Compared with the SAA+I/R group, p-ERK1/2 protein expression level was downregulated in the SAA+I/R+siRNA-DUSP4/16 group, which showed the other pathway (DUSP2) could be activated to inhibit ERK1/2 phosphorylation with SAA+ siRNA-DUSP4/16 during I/R. Compared with the SAA group, the p-JNK expression level was marked high in SAA+siRNA-DUSP4/16 group,which further demonstrated p-JNK was inhibited via DUSP4/16 with SAA pretreatment during I/R.

In Figure 8c, the results had shown that the activation of p-ERK1/2 was increased with siDNA-DUSP2 being added during I/R, which demonstrated DUSP2 could inhibit the activation of p-ERK1/2. In SAA+I/R group, p-ERK1/2 protein expression level was upregulated when siDNA-DUSP2 was added, which showed SAA could activate p-ERK1/2 by inhibiting DUSP2. In addition, during the course of I/R, the expression level of p-JNK was almost invariable with si-DUSP2 in use, thereafter apparently reduced with SAA pretreatment. Meanwhile, compared with the SAA+I/R group, p-JNK protein expression level was downregulated in the SAA+si-DUSP2+I/R group,above-mentioned results had demonstrated DUSP2 was downstream of JNK pathway, while DUSP2 was inhibited, ERK1/2 pathway was activated by negative-feedback, SAA could activate ERK1/2 to inhibit the activation of JNK by DUSP2 inhibitor.

To summarize, our data demonstrates that SAA exerts anti-apoptotic effects during myocardial IRI via the activation of ERK1/2 and inhibition of JNK, which results in increased Bcl-2 and reduced Bax, caspase 3 protein expression levels. Our results provide important insights into the understanding of the potential mechanisms involved in the cardioprotective effects of SAA. Further studies are needed to elucidate the exact mechanism by which SAA is protects the heart against IRI.

## Conclusions

**Figure 9:**
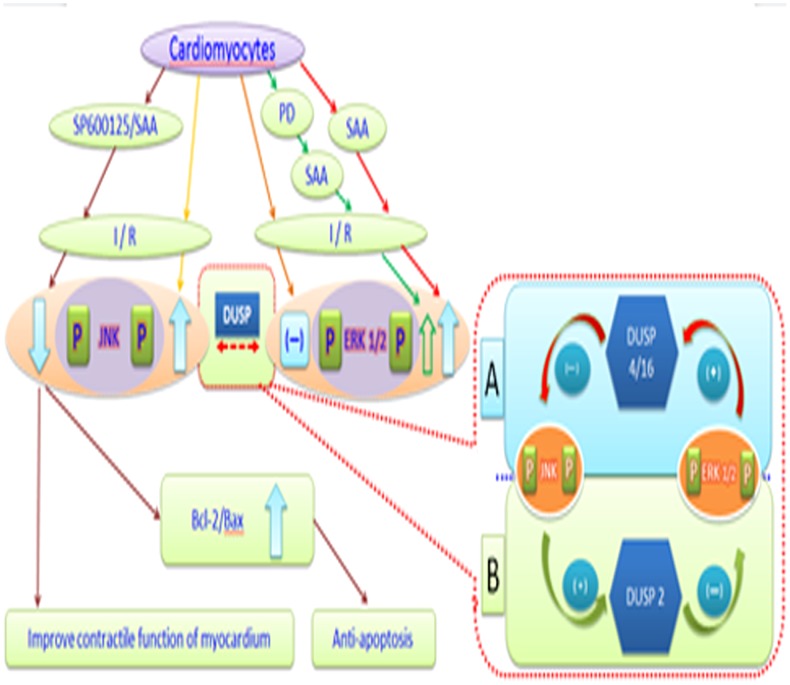
The possible mechanisms of SAA exerting its cardioprotective effects during I/R. SAA could play anti-apoptosis effect from myocardial IRI via the activation of ERK1/2 and inhibition of JNK, which resulted upregulation of ERK1/2 and downregulation of JNK, increased Bcl-2 and reduce Bax protein expression. JNK could inhibit the activation of ERK1/2 by DUSP2 mediating dephosphorylation of ERK1/2, and ERK1/2 mainly inhibits JNK activity by DUSP4/16-mediated dephosphorylation of JNK. SAA could activate ERK1/2 by inhibiting DUSP2-mediated JNK and inhibit JNK by activating DUSP4/16-mediated ERK1/2 to play anti-apoptosis from myocardial IRI.

JNK can inhibit the activation of ERK1/2 by DUSP2-mediated dephosphorylation of ERK1/2, while ERK1/2 mainly inhibits JNK activity by DUSP4/16-mediated dephosphorylation of JNK. SAA can activate ERK1/2 by inhibiting DUSP2-mediated JNK dephosphorylation and inhibit JNK by activating DUSP4/16-mediated phosphorylation of ERK1/2 to exert anti-apoptotic effects during myocardial IRI (see Figure 9).
